# An *in vitro* Evaluation of the Effect of Transient Electromagnetic Fields on Pacemakers and Clinical Mitigation Measures

**DOI:** 10.3389/fcvm.2020.607604

**Published:** 2020-12-23

**Authors:** Jing Huang, Kaibin Lin, Wu Lu, Ranran Ding, Bingjie Wu, Mingqi Cai, Saman Nazarian, Wenbin Zhao, Jingbo Li, Dong Huang

**Affiliations:** ^1^Department of Cardiology, Shanghai East Hospital, Shanghai Tongji University School of Medicine, Shanghai, China; ^2^Department of Cardiology, Shanghai Jiao Tong University Affiliated Sixth People's Hospital, Shanghai Jiao Tong University School of Medicine, Shanghai, China; ^3^College of Electrical Engineering, Shanghai University of Electric Power, Shanghai, China; ^4^Section for Cardiac Electrophysiology, Department of Medicine/Cardiology, University of Pennsylvania Perelman School of Medicine, Philadelphia, PA, United States

**Keywords:** pacemaker, intertwining pattern for pacing leads, electromagnetic susceptibility, cardiovascular implantable electronic devices, transient electromagnetic fields, electromagnetic interference

## Abstract

**Background:** The effect of transient electromagnetic fields on the function of pacemakers is not well-evaluated. There is a lack of effective methods for clinicians to reduce electromagnetic susceptibility (EMS) during implantation of pacemakers. This study aimed to evaluate whether a novel method of handling the excess leads in the pocket can lower the EMS of pacemakers and consequently reduce the effect of electromagnetic interference caused by transient electromagnetic fields on pacemakers.

**Methods:** An *in vitro* chest model was established to simulate the clinical condition of dual-chamber pacemaker implantation. Three different intertwining patterns of excess leads were examined: parallel, twisted once, and multiple twisted-pair. Oscillated currents were injected into a copper electrical wire set horizontally above the model to create a radiated magnetic field to simulate the transient daily electromagnetic exposure of pacemakers. The electromagnetic induction of current was measured. The occurrence of EMS-related adverse events was evaluated when the induced pulsed voltage was applied.

**Results:** Transient electromagnetic fields can induce electromagnetic noise in the pacing loop and inhibit the release of pacing pulses. The multiple twisted-pair intertwining pattern of excess leads was associated with a lower induced voltage amplitude than both the parallel and once-twisted patterns (*P* < 0.001). Even once twisted could significantly reduce induced voltage amplitude compared to not twisted (*P* < 0.001). A lower incidence of pacing inhibition was also observed in the multiple twisted-pair group than in the other two groups (*P* < 0.001).

**Conclusions:** Transient electromagnetic fields can cause pacing inhibition. Twisting the excess leads for multiple turns in the pocket is an effective method to reduce the EMS of the dual-chamber pacemaker.

## Introduction

With the improved worldwide life expectancy, cardiovascular implantable electronic devices (CIEDs) are increasingly common in patients with cardiovascular comorbidities (1). CIEDs are increasingly used in clinical practice as a result of the expanding indications for bradycardia, heart failure, and sudden cardiac death preventions (2). Electromagnetic interference (EMI) is an inevitable concern for implanted electronic devices when they are exposed to electromagnetic radiation. Typical radiation sources include charging and discharging equipment in daily life, such as mobile phones, household appliances, and power cables (3). In healthcare facilities, magnetic resonance imaging (MRI) and electrosurgery are common sources for possible EMI with CIED function (4). Patients with CIEDs are advised to avoid EMI as much as possible to prevent potential risks. The risks of EMI between CIEDs and radiation sources at the ubiquitous power frequency (50/60 Hz) and communication frequencies are well-studied (5). Transient electromagnetic fields formed by the power system transient processes are common in daily, occupational, and clinical conditions, which are initiated by the tripping, switching, and short-circuiting of electrical appliances. Such transient electromagnetic fields typically last on the order of micro- to milliseconds. Unlike the EMI generated by electrical appliances that work in 50/60-Hz power frequency or the EMI generated by mobile phones in communication frequencies, e.g., 950 MHz, the transient EMI is in the low-frequency range. According to the skin effect (6), this type of EMI carries sufficient energy and has sufficient penetration depth to pass through the pacing loop and may induce electromagnetic noise in the pacing loop and interrupt the CIED function. However, the effects of transient electromagnetic fields on CIEDs are not well-understood.

Although patients with CIEDs are generally at low risks of severe adverse EMI events (7), once serious EMI occurs, it may have severe consequences. EMI-induced oversensing may cause inappropriate mode switch (DDD→DDI/VVI), unnecessary ventricular pacing, asynchronous pacing (VOO), electrical reset, pacing inhibition, and inappropriate implantable cardioverter defibrillator (ICD) therapy (anti-tachycardia pacing or shock) (8, 9). Reducing the chance of malfunction caused by EMI is important for pacemaker manufacturers, engineers, and physicians. In addition to tremendous industry efforts such as the production of MRI conditional systems (10), it would be very helpful if clinicians can reduce the electromagnetic susceptibility (EMS) of CIEDs by altering the implantation technique of pacemakers.

The use of bipolar leads with minimal tip-to-ring spacing, optimized sensitivity settings, optimal lead-tip position, and orientation play important roles in reducing the susceptibility of CIEDs to exogenous EMI (7, 9, 11). However, the optimal method to handle excess leads in the subcutaneous or submuscular pocket has not been reported. Excess atrial and ventricular lead sections of varying length are routinely left in the pocket during the implantation procedure. The twisted-pair pattern is widely used in power transmission systems due to its ability to reduce the EMS (12). This pattern is formed by two copper wires insulated from each other and twisted into a helical structure with multiple turns. In this study, we sought to examine the effects of the twisted-pair pattern on the electromagnetic compatibility (EMC) of pacemakers. For the very first time, this novel twisted-pair intertwining pattern to handle excess pacing leads is tested *in vitro* to evaluate its effect on the EMS with dual-chamber pacemakers. This twisting method to handle excess pacing leads provides a novel and simple approach for clinicians to reduce the risk of EMI for CIEDs without high costs.

## Methods

This *in vitro* study was approved and monitored by the Institutional Animal Use and Care Committee at Shanghai Jiao Tong University Affiliated Sixth People's Hospital and Shanghai University of Electric Power and conformed to the Guide for the Care and Use of Laboratory Animals. It was implemented at the High Voltage Laboratory in Shanghai University of Electric Power (Shanghai, China). EMC compliance and proper safety earthing were observed during the tests.

### Simulation Model

Dual-chamber pacemakers employed in this study were manufactured by Medtronic (Model: Relia RED01, Minneapolis, US), Vitatron (Model: C50D C50A3, Kanaalweg, NL), and St. Jude Medical (Model: AccentDR PM2112, St. Paul, US). Active fixation bipolar pacing leads were used (Medtronic 5,076–52 cm for atrial and Medtronic 5,076–58 cm for ventricular). Before the tests, all pacemakers were interrogated to ensure adequate battery voltage and function. The sensitivity was set at 2.5 mV for the ventricle and 0.5 mV for the atrium, and both pacing outputs were set at 2.5 V/0.4 ms. Both leads were programmed to the bipolar mode. The pacing mode was programmed to be DDD at a rate of 50–60 bpm (1,000–1,200 ms).

**Figure 1:**
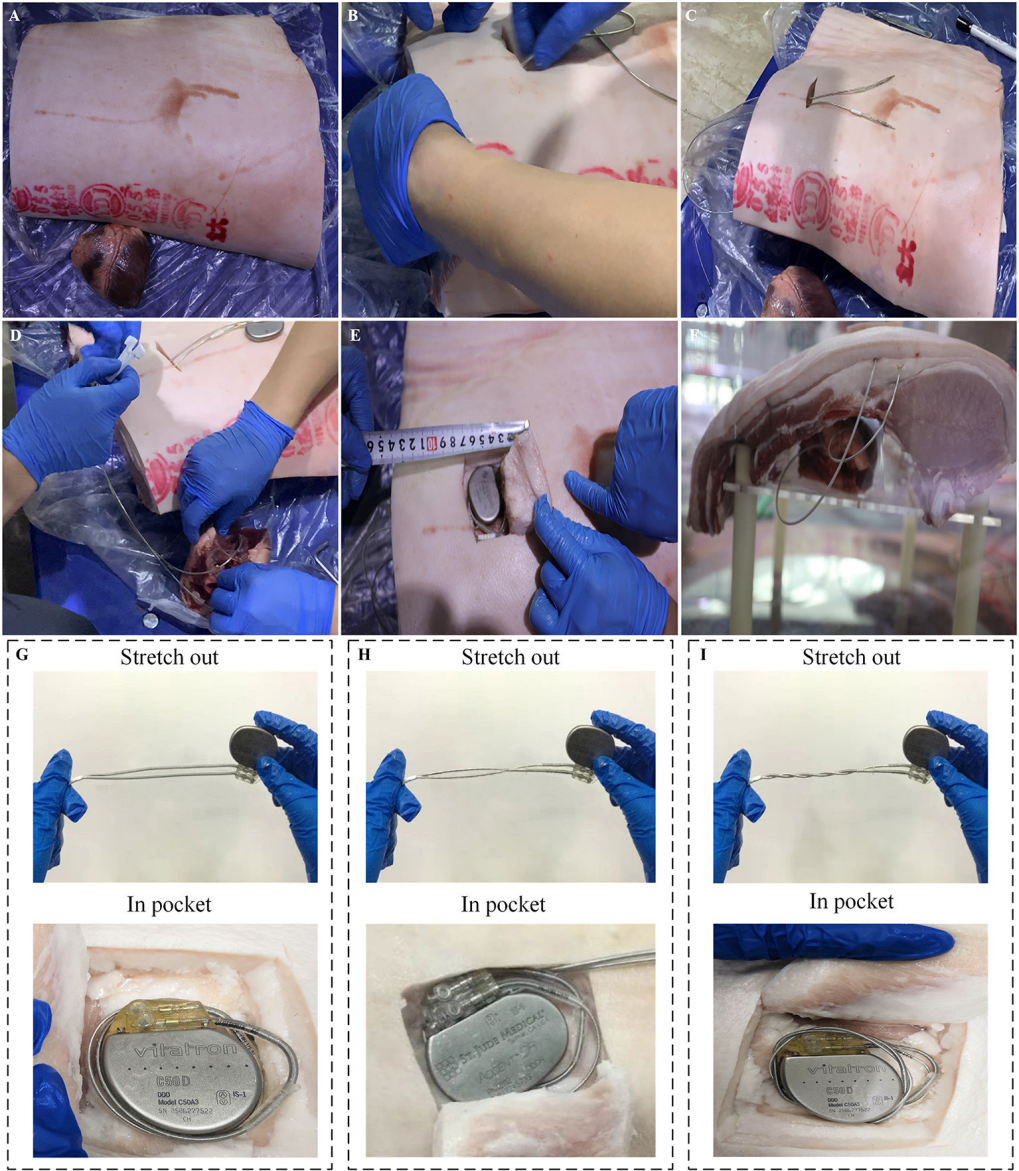
Experimental setups of an artificial human chest model. **(A)** Swine rib and heart. **(B,C)** Process of atrial and ventricular leads passing through a subcutaneous tunnel. **(D)** The atrial and ventricular leads were fixed at the right atrial appendage and right ventricular apex, respectively, which simulated the procedure of lead implantation in clinical conditions. **(E)** The excess atrial and ventricular leads were coiled up and put into the pocket with the pacemaker. **(F)** The human chest model was immersed into a cylindrical Perspex container filled with 0.9% saline solution. **(G–I)** Illustration of three types of intertwining patterns: parallel arrangement, once twisted and multiple twisted as a twisted pair.

As shown in Figure 1A, a swine torso with skin and rib cage was used to simulate the anatomical structure of the chest wall for a human body, and the swine heart was used to simulate the anatomical structure of a human heart. The swine ribcage and heart were oriented with relative position mirroring the human orientation. The atrial and ventricular leads were passed through a subcutaneous tunnel and fixed at the right atrial appendage and right ventricular apex, respectively (Figures 1B–D). The excess atrial and ventricular leads were coiled into the pocket with the pacemaker (Figure 1E). After implantation, the chest model was placed in a cylindrical Perspex container filled with ~85 L of 0.9% saline solution. The electrical resistance of saline solution was adjusted to 300–800 ohms to simulate the internal environment of a human body, as shown in Figure 1F (13).

### Transient Electromagnetic Field Generation and Measurement

**Figure 2:**
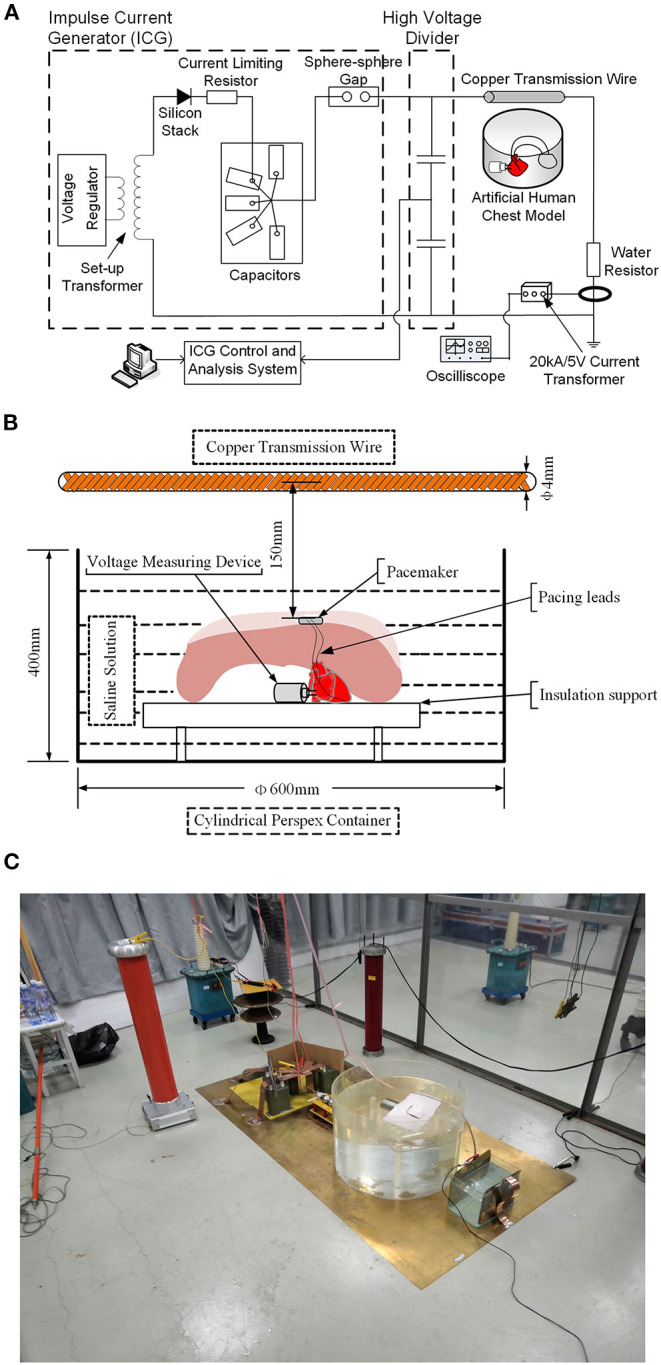
Sketch of test setups to generate the transient electromagnetic field. **(A)** Schematic diagram of the test circuit. **(B)** Schematic drawing of the relative positions of the human chest model and copper transmission wire. **(C)** Physical profiles of the test circuit.

An impulse current generator was used to produce a damped oscillating current waveform. The details of experimental setup are shown in Figure 2. A setup transformer (220 V/5 kV) and a voltage regulator were used to produce DC input voltages. The DC voltages were passed through a high-voltage silicon rectifier stack (100 kV/1A) and a current limiting resistor and charged into a group of capacitors (1 μF) with a water resistor (50 ohm) in series for damping purposes. Followed by breaking a sphere–sphere gap in series, the transient current waveform was eventually formed. The damped oscillating current waveforms have amplitudes of 1–5 kA and an oscillating period of approximate 20 μs, and an example of the current waveform is shown in Supplementary Figure 1. The transient impulse current is injected into a standard copper transmission wire with a diameter of 4 mm. The copper wire was set horizontally 150 mm above the pacemaker in the pocket, as shown in Figure 2B. Using the electromagnetic field theory, the amplitude of the magnetic field generated by the transient current on the pacing loop was calculated to be ~1.3–6.5 mT.

**Figure 3:**
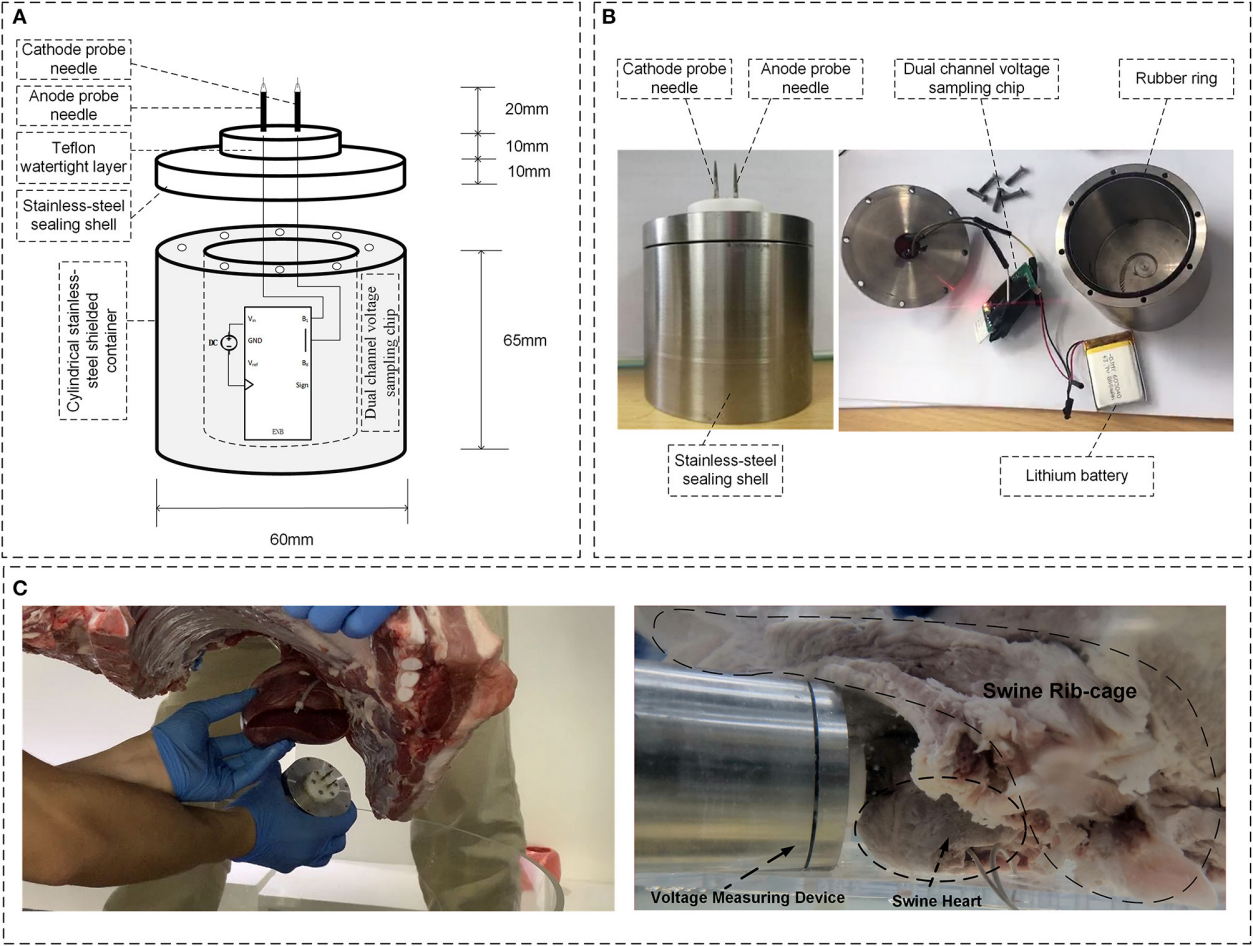
Overview of the voltage-measuring device. **(A)** Inboard profile of the device. **(B)** Physical profile of the device; **(C)** Implantation of the voltage-measuring device into the myocardium.

Since the induction effect caused by the transient electromagnetic fields may permanently damage the pacemaker programmer, and the programmer may not be able to record the induced EMI signals with frequency higher than 1 kHz, a custom broadband voltage-measuring device was designed to record the electrical signals, including those produced by the normal pacemaker function and pulsed high-frequency voltage induced by the transient electromagnetic fields. The inboard and physical profiles for the device are shown in Figures 3A,B, respectively. The voltage-measuring device has a cylindrical shell made by stainless steel for the purpose of magnetic shielding. A pair of anode and cathode needle electrodes was inserted on top of the device. Needle electrodes were connected to an analog-to-digital signal-sampling printed circuit board (PCB) to measure the voltage signals with frequency up to 500 kHz. The implantation of the voltage-measuring device is shown in Figure 3C. Using a Teflon watertight intermediate layer and proper sealing, waterproof performance was attained. Once implanted, the voltage-measuring device function was continually maintained for 1 h using a lithium battery, and >5-G data could be recorded using a secure digital memory card.

### Study Protocol

Three different intertwining patterns of excess atrial and ventricular leads were tested on the *in vitro* chest model: parallel arrangement, once twisted, and multiple twisted (10 times) as a twisted pair. The schematic and physical profiles for the three types of intertwining patterns are shown in Figures 1G–I. The amplitude of the impulse current was increased from 1 to 5 kA in 1-kA steps. A set of 10 induced voltage measurements was performed for each impulse current level. The changing trends of the amplitude of induced voltages in the pacing loop with the increase in impulse current were recorded and compared among the intertwining patterns of excess leads and pacemaker conditions. Furthermore, all EMI-related adverse events were carefully recorded, including the possible electrical reset, pacing inhibition, and pacemaker malfunction.

### Statistical Analysis

All statistical analyses were performed using SPSS 26.0 (SPSS Inc., Chicago, IL., USA). The data are presented as the mean values ± standard deviation. Kolmogorov–Smirnov test was used to check the normality of the quantitative data. Levene's test was used to assess the variance homogeneity. The variance analysis was applied for comparisons of homoscedasticity data. The Kruskal–Wallis test was applied for comparisons of heteroscedasticity data. Qualitative data were compared using the χ^2^-test. *P* < 0.05 was considered statistically significant in all analyses.

## Results

### Effect of the Amplitude of the Impulse Currents on the Risk of Electromagnetic Interference

**Figure 4:**
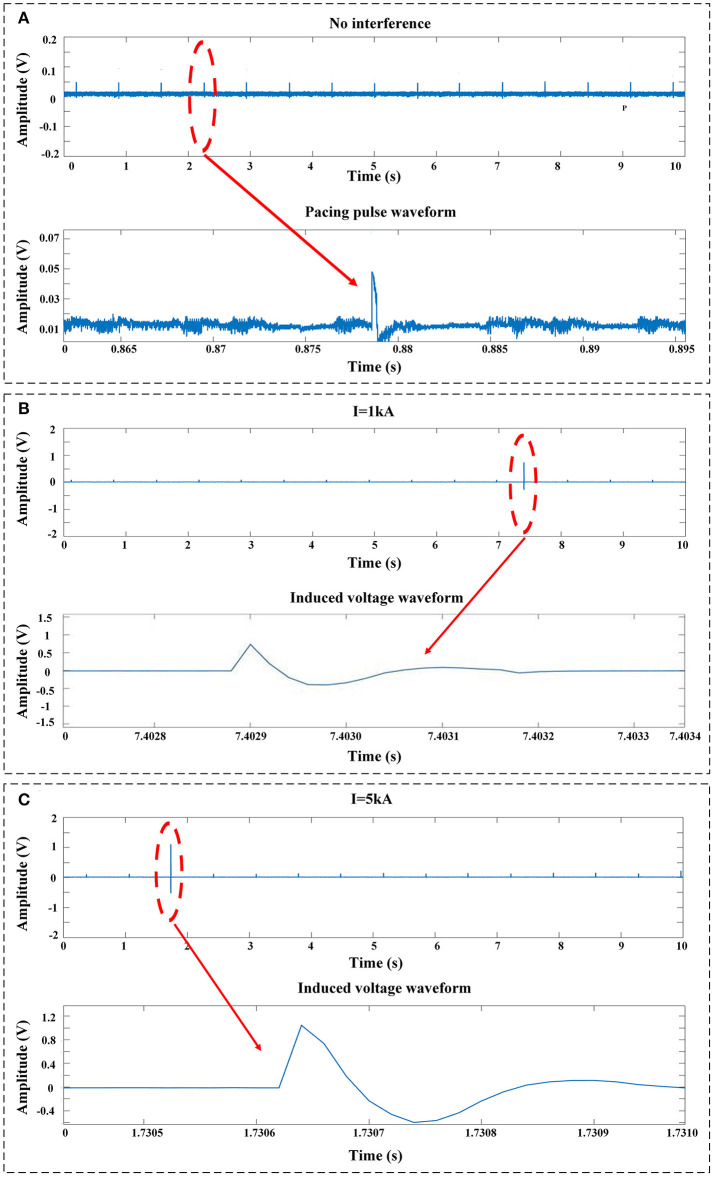
Typical signals recorded by the voltage-measuring devices. **(A)** No impulse current was applied. The signals were the pulses released by the pacemaker. **(B)** EMI signals measured at an impulse current of 1 kA. **(C)** EMI signals measured at an impulse current of 5 kA.

Figure 4A shows the typical pacing pulse signals recorded by the voltage-measuring devices when no impulse current was applied. Normal pacing pulse signals on the surface of the myocardium initiated by the pacemaker are regular pulsed signals with a pacing rate of ~50~60 bpm and a magnitude of ~40~50 mV. Once the impulse current is applied, typical EMI signals obtained by the voltage-measuring devices are shown in Figures 4B,C. The EMI signals are induced voltages in oscillating decay-type shapes; the intensity of EMI is defined as the maximum oscillation amplitude on the waveform, i.e., the first crest that appears on the waveform. As shown in Figure 4, the intensity of EMI gradually increases with the increase in impulse current amplitude, i.e., the intensity of EMI at an impulse current of 5 kA is ~2~3 times higher than that at an impulse current of 1 kA (*P* < 0.001). In all cases where the impulse current is applied, the induced voltage is higher than the sensitivity setting of 2.5 mV for the ventricle and 0.5 mV for the atrium.

### Effect of the Intertwining Pattern of the Excess Leads on the Risk of Electromagnetic Interference

**Figure 5:**
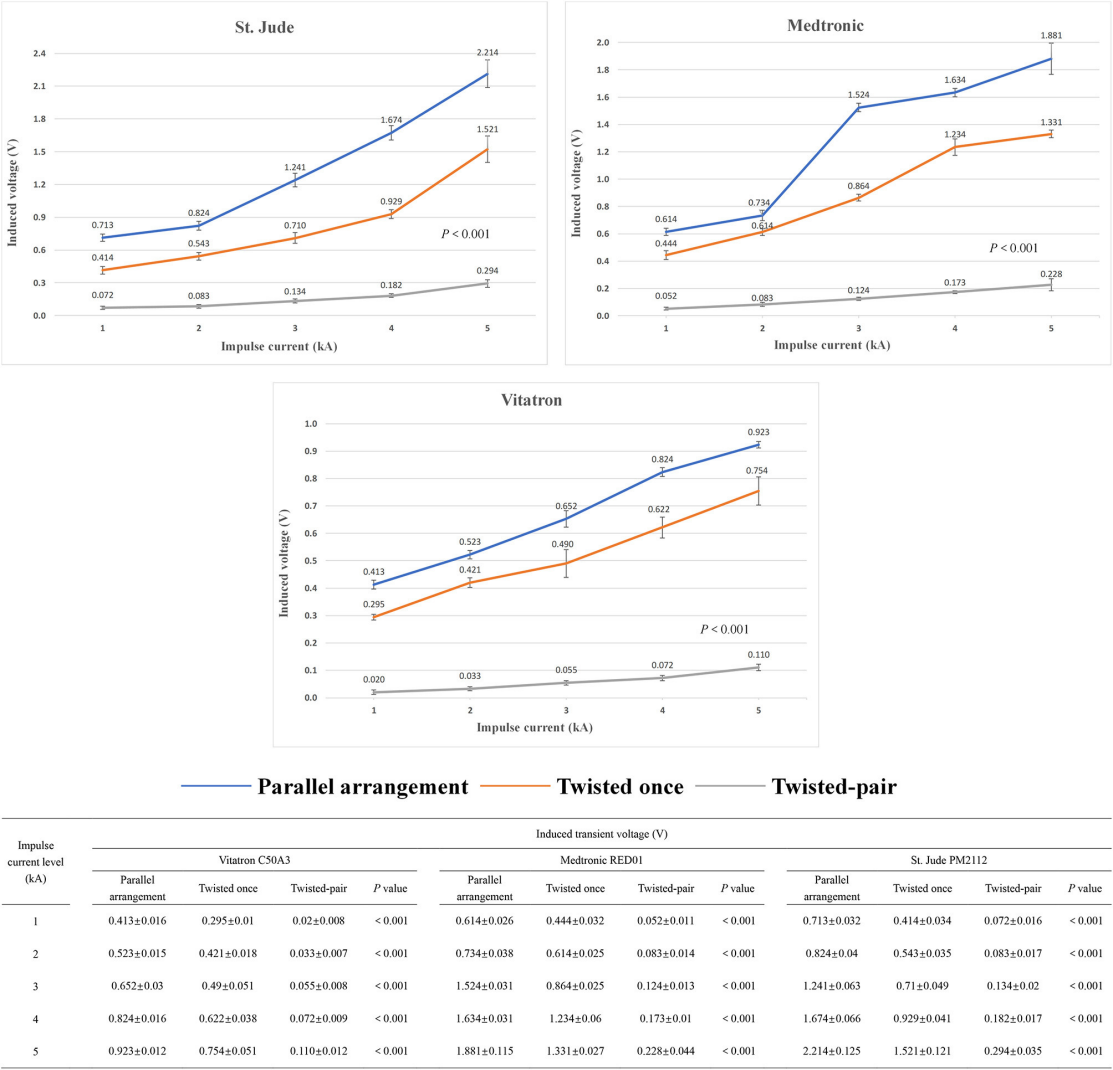
Changing trend and measured value of the induced voltage in the pacing loop generated by the increased impulse current with different intertwining patterns and pacemaker manufacturers.

Figure 5 shows the relationship between induced transient voltages and impulse current levels with different intertwining patterns of the device manufacturer and excess lead. The mean values and standard derivations of the induced voltage under all test conditions are summarized in Figure 5. At a constant impulse current level, the twisted-pair intertwining pattern for the excess pacing leads is obviously associated with a significant reduction in amplitude of the induced voltage compared to the parallel and once-twisted arrangements for the excess leads (*P* < 0.001). Once-twisted is also better than the parallel pattern in reducing the induced voltage (*P* < 0.001). Besides, compared with the parallel pattern, the twisted-pair intertwining pattern and once-twisted pattern, respectively, reduce the amplitude of induced voltage by ~90% and 20–40% for all pacemakers enrolled in the study. No significant effect of any particular pacemaker manufacturer was noted.

### Effect of the Intertwining Pattern of Excess Leads on the Risk of Pacing Inhibition

**Figure 6:**
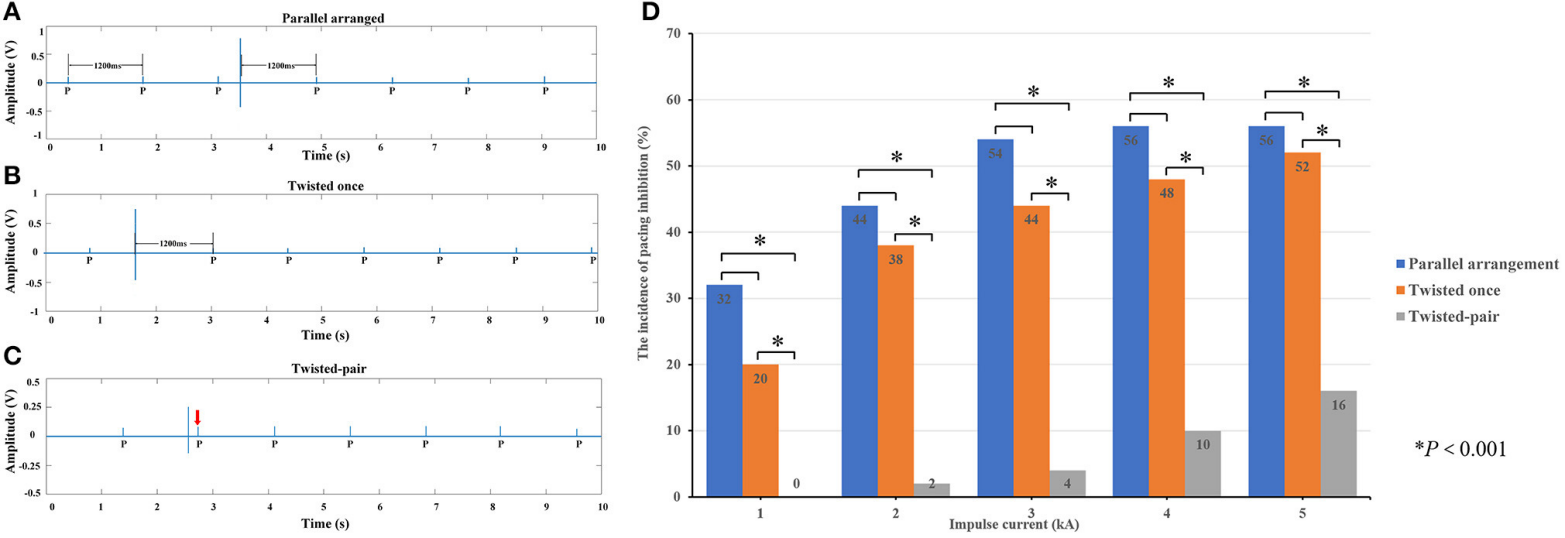
Pacing inhibition events observed in the study. **(A)** When the excess leads were in the parallel arrangement, the induced voltage generated by an impulse current inhibited the pacing. The escape interval was equal to the pacing interval (1,200 ms). **(B)** When the excess leads were twisted once, the amplitude of induced voltage decreased, but the pacing remained inhibited. **(C)** When the excess leads were oriented in the twisted-pair configuration, the amplitude of induced voltage significantly decreased, and the pacing was not affected (where the red arrow is pointing). **(D)** Incidence of pacing inhibition depending on the intertwining pattern of the pacing leads.

Since the amplitude of the induced transient voltage is higher than the typical sensitivity settings of pacemakers, there is a risk of harmful pacing inhibition events due to oversensing of the induced voltage. A typical example of the pacing inhibition event observed by the voltage-measuring device is shown in Figure 6A. By changing the intertwining pattern of excess leads from parallel arrangement to the once-twisted arrangement, the evidence of pacing inhibition remains, as shown in Figure 6B. However, with the multiple twisted-pair arrangement, the pacing inhibition disappears, as shown in Figure 6C. The enhanced ability of pacemakers against pacing inhibition by using the twisted-pair pattern for excess leads is readily observed by comparing the incidence of pacing inhibition for all configurations, as shown in Figure 6D (*P* < 0.001). The incidence of pacing inhibition also showed the trend to be decreased when the leads were twisted once compared to the parallel arrangement, but it was not statistically significant. Furthermore, evidence of EMI-related adverse events such as asynchronous pacing, electrical reset, and device malfunction was not observed during the study.

## Discussion

In this study, we first found that twisting the excess pacing lead that remained in the pocket to form the twisted-pair pattern could reduce the EMS of a dual-chamber pacemaker in transient electromagnetic fields.

It is well-accepted that CIEDs are susceptible to strong electromagnetic fields (EMFs) (14). The enhanced EMC of CIEDs is a common challenge for patients, engineers, and clinicians to face together. Patients with CIEDs are warned to avoid being exposed to all abnormal electromagnetic fields, particularly MRI scan, where the magnetic field strength is 1.5–3.0 T. Over the past few decades, efforts were made on the CIED hardware design to decrease the EMI by various means such as using a Hall sensor to replace the reed switch, applying circuit protection to lithium-iodine cells, minimizing the ferromagnetic elements, adding a filter capacitance, and using bipolar leads. Consequently, modern generations of CIEDs are no longer absolute contraindications to strong electromagnetic fields (EMF), even facing MRI scan (15). However, potential interaction of the EMI with CIEDs remains possible (16, 17). The oversensing-induced pacing inhibition is the most relevant problem in clinical cases (18). It is necessary to set the pacing mode to asynchronous pacing (VOO) before an exposure to strong EMFs.

In daily, occupational, and clinical conditions, the main EMFs are in the power frequency range of 50/60 Hz. Previous publications indicate that pacemakers with bipolar sensing are less likely affected by EMFs in the power frequency (8, 9). However, some instances of EMI may occur during daily activities, which makes our current understanding of their effects on the pacemaker function underestimated. EMF sources in power frequency such as power lines, household appliances, and many types of electric devices are not always working at steady state. Power system transient processes such as short circuit, tripping, and switching operation of circuits can form impulse currents with an instantaneous strong radiated EMF. These transient processes are common for all the devices with charging and discharging functions in daily, occupational, and clinical conditions. To the best of our knowledge, the effects of transient electromagnetic fields on the pacemaker function have not been evaluated. The impulse current caused by the power system transient process is approximately in the kA range, and the formed strength of radiated magnetic fields ranges from mT to T (7). The frequency of the EMI under these circumstances is generally higher than 1 kHz. A systematic review revealed that CIEDs were susceptible to malfunction induced by EMF in the kHz–MHz range (19). In the present study, impulse currents with amplitudes in the order of kA and duration in the order of μs were generated to simulate the power system transient processes in the real world. Typical electromagnetic exposure scenes with similar magnetic field intensities and interaction ranges are presented in Supplementary Table 1. Notably, the EMI related to the power system transient process may result in oversensing events and pacing inhibition, which may be harmful to pacing-dependent patients, and if this type of EMI persistently exists, it may cause catastrophic consequences such as cardiac arrest and sudden death. Because the oversensing events may be short-lived, they are difficult to be retrospectively diagnosed by the programmer; therefore, the power-system-transient-process-induced EMI may occur more frequently than clinically appreciated.

To reduce EMI events related to power system transient processes, a novel method to handle the excess leads is presented in this study. To the best of our knowledge, to date, there are few studies on the strategies for clinicians to decrease EMI effects on CIEDs through improvements in the implantation procedure. Only one recent study suggested that more horizontal orientation and a medial position of the atrial and ventricular lead tip could reduce the risk of EMI for pacemakers (11). During implantation, almost all proceduralists are accustomed to make the excess atrial and ventricular leads parallel with each other and connected to the pacemaker. In the following implantation step, the excess leads are coiled up and put into the pocket together with the pacemaker. In the present study, we note that the parallel arrangement for excess leads creates a greater area for electromagnetic induction, which is undesirable for EMC. Referencing the usage of the twisted-pair pattern in power transmission lines with excellent anti-interference ability, the present study verified *in vitro* via an artificial human chest model that the twisted-pair intertwining pattern of excess pacing leads could effectively decrease the induced voltage in the pacing loop and reduce the risk of EMI related oversensing events.

**Figure 7:**
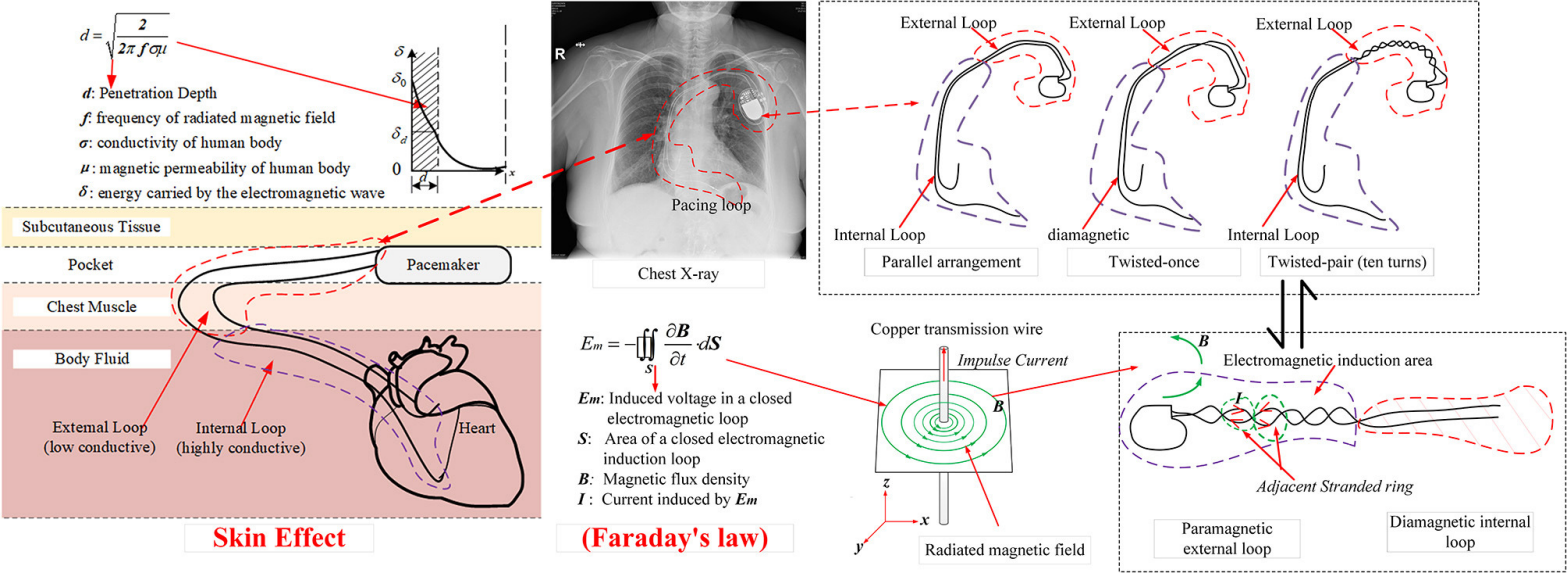
Schematic illustration of the mechanism to form an EMI signal due to the electromagnetic induction.

When the copper transmission wire is charged with an impulse transient current, a radiated magnetic field with the central axis of copper wire as the center of radiation is formed, as shown in Figure 7. The oscillating frequency of the magnetic field is ~50 kHz, i.e., this magnetic field is a low-frequency time-varying electromagnetic signal. From the view of a time-varying electromagnetic field, due to the skin effect (6), the penetration depth of the radiated magnetic field in the simulated *in vitro* model is given by

(1) $$\begin{array}{l} \text{d}=\sqrt{\frac{1}{\pi \text{f}\sigma \mu }} \end{array}$$

where *f* is the frequency of the magnetic field, Hz; σ is the conductivity of the simulated model, S/m; and μ is the magnetic permeability of the simulated model, H/m. For dual-chamber pacemaker implantation, the pacing loop enclosed by the leads can be divided into two sections: internal pacing loop enclosed by internal leads, which extend to the right atrium and right ventricle, and external pacing loop enclosed by the excess leads left in the pocket. Since the internal leads are deeply immersed in blood and human tissue, the internal loop is highly conductive with a conductivity of ~4 S/m (20, 21). In contrast, the external leads are immersed in the shallow pocket, the surrounding environment is quite dry, and the external loop has a low conductivity of 0.015~0.15 S/m (20, 21). Furthermore, both loops have approximately identical magnetic permeabilities to that in air (20). According to formula (1), the penetration depth in internal loop is minimum, i.e., the flux of the radiated magnetic field cannot pass through the internal loop. The internal loop makes no contribution to the magnetic induction. The flux of the radiated magnetic field can pass through the external loop and initiate the transient induced voltage, i.e., the EMI signal. According to Faraday's law, the amplitude of transient induced voltage initiated in the external loop is given by

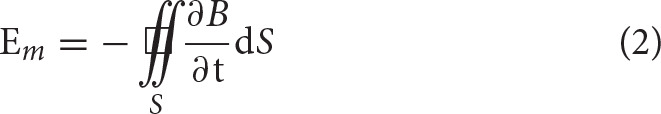



where *B* is the magnetic flux density, T; *S* is the effective electromagnetic induction area of the external loop, m^2^. According to formula (2), for the same magnetic flux density produced by the low-frequency time-varying magnetic field, the amplitude of induced voltage is proportional to the effective electromagnetic induction area of the external loop. As shown in Figure 7, by using the twisted-pair pattern for excess leads, the magnetic inductions formed in adjacent stranded rings are neutralized, which results in a much smaller effective electromagnetic induction area than that when the leads are in the parallel arrangement. Consequently, the amplitude of the induced transient voltage is reduced by ~90%.

During the pacemaker implantation process, it is not difficult to make the excess atrial and ventricular leads twisted with each other. We have attempted this novel intertwining pattern in several patients (Supplementary Figure 2). One must emphasize that the tension of the leads must not be excessive, and the suture sleeves must be tightly sutured to the pectoral muscle to avoid the lead-tip dislocation. Additionally, when twisting prior to connecting, the atrial and ventricular leads must be carefully differentiated before being inserted into the appropriate pacemaker ports. During the period of 1-year follow-up, the pacing threshold, sensing, and especially impedance were stable. No abnormal functioning of pacemakers with these novel intertwining patterns have been observed. In general, this novel method of handling the excess leads is safe and feasible.

Finally, experimental results in this study indicate that the power system transient processes do not appear to cause serious malfunctions with the pacemaker. Oversensing is the main problem caused by such EMI. Therefore, after device implantation, particularly for pacing-dependent patients, avoidance of switching operations of high-power electrical appliances is recommended.

Our study has some limitations. First, this is an *in vitro* study. Although we tried our best to simulate real-world scenarios, differences from real-world environments remained. In our study, the distance between the electric transmission wire and the simulation model was ~15 cm, which simulates an extreme distance between patients and instantaneous current sources. In actual conditions, this distance should be kept as far as possible to minimize the effect of the transient EMI on pacemakers. Additionally, the complex internal environment of the human body and clinical implantation process of ventricular and atrial leads cannot be strictly repeated. Second, the swine heart model does not generate its intrinsic impulses and mechanical contraction. For future work, an *in vivo* animal experiment is necessary to further verify the effect of twisted pair intertwining pattern of leads on EMS. Third, a dual-chamber pacemaker system was the only type of CIEDs under investigation in this study. Further assessments are necessary to verify whether the present research results can be generalized to ICD or biventricular systems. Fourth, lead twisting will make lead extraction difficult. Finally, the twisting pattern of the leads may increase the risk of lead dislodgement during the acute phase of the implant and may cause lead abrasion in the long term. Twisting too many times may cause excessive tension and more likely damage the pacing lead. According to the results of this study, the induced voltage in the pacing loop is significantly reduced even when the lead was only once twisted. Pacing inhibition events also showed the trend to be decreased when the leads twisted. It suggested that we do not have to twist too many times; twisting not more than three times can also reduce the EMS of the pacing loop and may avoid the risk of lead damage. Further *in vivo* animal study has been scheduled, and we plan to compare the abrasion of leads with different twisting turns over time to produce more convincing evidence. Whether the pattern of the leads shows superiority in clinical practice warrants further clinical studies with the appropriate sample size and follow-up.

In conclusion, power system transient processes such as short circuit, tripping, and switching operations of typical electric devices can generate impulse currents with a strong radiated EMF. This EMF may induce oversensing in pacemakers and inhibit pacing. Patients who receive CIEDs should be informed to keep away from switching operations of electrical equipment. Furthermore, by twisting the excess atrial and ventricular leads for several turns to form the twisted-pair pattern during the process of pacemaker implantation, the EMC is improved, and the susceptibility to pacing inhibition is reduced.

## Data Availability Statement

The original contributions presented in the study are included in the article/**Supplementary Materials**, further inquiries can be directed to the corresponding author/s.

## Ethics Statement

The animal study was reviewed and approved by the Institutional Animal Use and Care Committee at Shanghai Jiao Tong University Affiliated Sixth People's Hospital and Shanghai University of Electric Power.

## Author Contributions

DH, WZ, and JL: conceptualization. JH and WL: study design and data analysis. JH, KL, WL, RD, BW, and MC: experiment implementation. JH, WL, and KL: writing—original draft. WZ, JL, and SN: writing—review & editing. DH: project administration. All authors contributed to the article and approved the submitted version.

## Conflict of Interest

The authors declare that the research was conducted in the absence of any commercial or financial relationships that could be construed as a potential conflict of interest.

## Abbreviations

EMS: electromagnetic susceptibility
EMI: electromagnetic interference
CIED: cardiovascular implantable electronic device
MRI: magnetic resonance imaging
ICD: implantable cardioverter defibrillator
EMC: electromagnetic compatibility
PCB: printed circuit board
EMF: electromagnetic field.
