# Supplementary material for: An in vitro Evaluation of the Effect of Transient Electromagnetic Fields on Pacemakers and Clinical Mitigation Measures
Source: Front Cardiovasc Med. 2020 Dec 23;7:607604. doi: 10.3389/fcvm.2020.607604 (PMC7785788; doi:10.3389/fcvm.2020.607604)
Supplement: Supplementary file 1 [file Data_Sheet_1.docx]

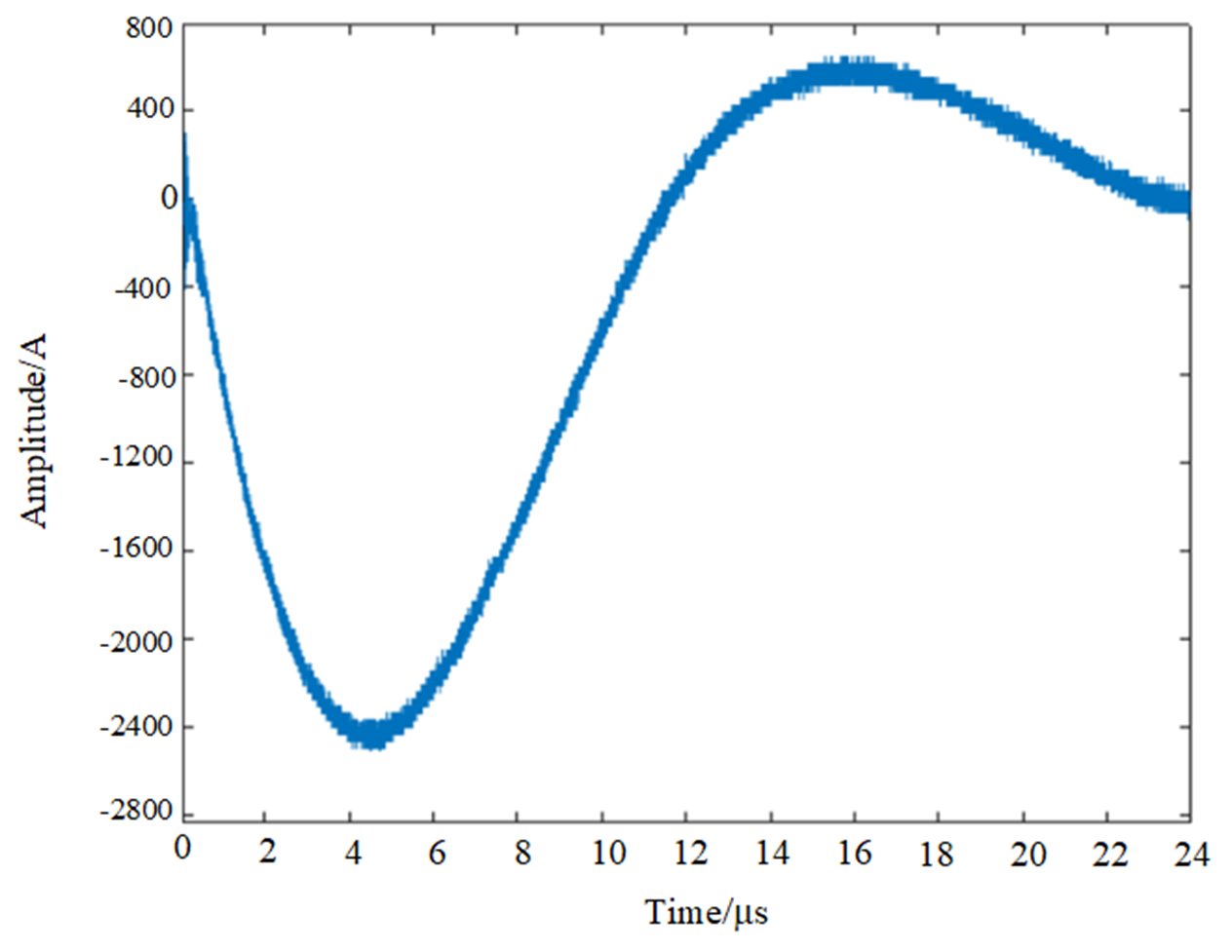


**Supplementary material Figure I.** A typical waveform of transient impulse current, the amplitude for the exampled current is approximately 2.5 kA.


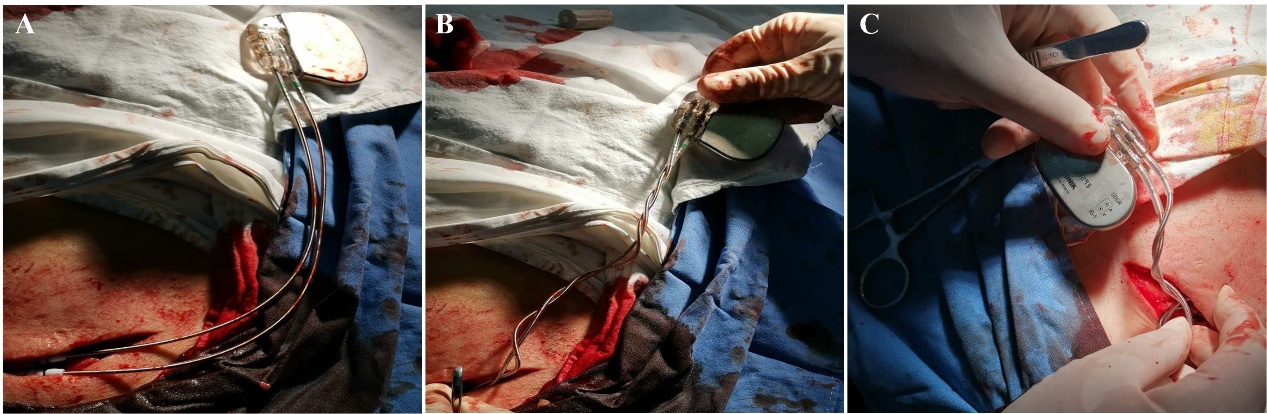


**Supplementary material Figure II.** The process of twisting the excess leads during clinical pacemaker implantation. (A) Conventional parallel arrangement of excess leads (B) The excess atrial and ventricular leads twisted with multiple turns as twisted-pair. (C) Put the twisted lead and pacemaker into the pocket.
