# Supplementary material for: An in vitro Evaluation of the Effect of Transient Electromagnetic Fields on Pacemakers and Clinical Mitigation Measures
Source: Front Cardiovasc Med. 2020 Dec 23;7:607604. doi: 10.3389/fcvm.2020.607604 (PMC7785788; doi:10.3389/fcvm.2020.607604)
Supplement: Supplementary file 2 [file Table_1.docx]

**Supplementary material Table I Typical electromagnetic exposure scenes having similar magnetic field intensities and interaction ranges as the impulse current**

| Environment |  | Source |  | Magnetic intensity |  | Typical distance |  | Pulse width/Frequency |
| --- | --- | --- | --- | --- | --- | --- | --- | --- |
| Daily life |  | Xenon light source |  | 0.02mT |  | 2m |  | ms |
|  |  | hairdryer |  | 2mT |  | 0.03m |  | ms |
|  |  | Electric shaver |  | 1.5mT |  | 0.03m |  | ms |
| Occupational  conditions |  | Cable maintenance（500A/ phase） |  | 1.3mT |  | 0.075m |  |  |
|  |  | On-current of three-electrode gas spark switch |  | 1.08mT |  | 1.5m |  | ms |
|  |  | Three-phase short-circuit fault of 110kV transformer |  | 1.86mT |  | 2.5m |  |  |
|  |  | lightning strikes to 35kV substation |  | 2.16mT |  | 3m |  | μs |
|  |  | Off-current of 225A welding |  | 1mT |  | 0.1m |  | μs |
|  |  | 1000A welding |  | 1.3mT |  | 0.2m |  |  |
| Medical  radiations |  | On-current of telemetry receiver |  | 2mT |  | 0.03m |  | 432MHz |
|  |  | Electrosurgical units |  | 0.99mT |  | 0.05m |  | 240KHz |
|  |  | Cavitron Ultrasonic Surgical Aspirator |  | 3.9*10^6T  （4.9A/m） |  | 0.1m |  | 23KHz |
